# NXT2 is required for embryonic heart development in zebrafish

**DOI:** 10.1186/1471-213X-5-7

**Published:** 2005-03-24

**Authors:** Haigen Huang, Bo Zhang, Parvana A Hartenstein, Jau-nian Chen, Shuo Lin

**Affiliations:** 1 Department of Molecular, Cell, and Developmental Biology, University of California, Los Angeles, California 90095, USA; 2 Center of Developmental Biology and Genetics, College of Life Sciences Peking University, Beijing 100871, P. R. CHINA

## Abstract

**Background:**

NXT2 is a member of NXT family proteins that are generally involved in exporting nuclear RNA in eukaryotic cells. It is not known if NXT2 has any function in specific biological processes.

**Results:**

A zebrafish mutant exhibiting specific heart defects during embryogenesis was generated by animal cloning-mediated retroviral insertions. Molecular analysis indicated that the mutant phenotype was caused by a disruption of *NXT2*. Whole-mount RNA *in situ *hybridization showed that *NXT2 *transcripts were clearly detectable in embryonic heart as well as other tissues. Further analysis revealed that expression level of one form of alternative splicing *NXT2 *mRNA transcripts was significantly reduced, resulting in deficient myocardial cell differentiation and the malformation of cardiac valve at the atrioventricular boundary. The defects could be reproduced by morpholino anti-sense oligo knockdown of *NXT2*.

**Conclusion:**

NXT2 has a critical role in maintaining morphogenetic integrity of embryonic heart in vertebrate species.

## Background

In a living eukaryotic cell, the nuclear envelope is a critical barrier between the nucleus and the cytoplasm, which prevents macromolecules from freely exchanging between the two compartments. The eukaryotic cells have developed multiple pathways designed to transport various types of macromolecules through specialized channels that are incorporated within the nuclear envelope. There are four major components involved in nucleocytoplasmic transport, including cargos, receptors, nuclear pore complex (NPC), and Ran [1-3]. Cargos are proteins, RNAs or ribonucleoprotein (RNP) particles that contain either nuclear localization signals destined for import into the nucleus from the cytoplasm or nuclear export signals destined for export from the nucleus to the cytoplasm [4-6].

NXF (nuclear export factor) family members (NXF 1 to 6) play essential roles in RNA exporting out of the nucleus [7,8] through NPC. The structure of the founding member, NXF1 (also called TAP), has modular organization consisting of four major domains: a noncanonical RNP-type RNA-binding domain (RBD), four leucine-rich repeats (LRRs), an internal domain sharing significant sequence homology with the nuclear transport factor 2 (the NTF2-like domain) and a C-terminal ubiquitin associated (UBA)-like domain. The RBD domain and LRRs are responsible for recognition and binding of RNAs through RNP and CTE (constitutive transport element-a cis-acting RNA sequence). The UBA-like domain interacts with multiple nuleoporins of NPC. The NTF2-like domain heterodimerizes with another protein, NXT1 [9,10].

NXT1 was initially identified from human based on its sequence homology to NTF2 (~26%) [11,12] and association with the RNA exporting factor NXF1/TAP [13]. Earlier studies suggested that NXT1 was necessary for Crm1-mediated nuclear export of proteins [14], in which NXT1 directly binds to export receptor Crm1 and RanGTP (a form of Ran binding GTP), and efficiently stimulates the release of cargos in the cytoplasm after translocation through NPC [15]. However, recent experiments demonstrated that NXT1 appears to exclusively participate, independently of Ran and Crm1, in TAP-mediated nuclear export of RNAs. This finding has been widely confirmed in yeasts, *Drosophila*, *C. elegans *and vertebrates [7,9,10,16-18]. It was shown that formation of a NXF1/NXT heterodimer via NTF2-like domain is required for the nuclear export of RNAs in a wide range of eukaryotic organisms [10,19,20]. Recently, another member, NXT2 (also called p15-2), was identified to be able to substitute NXT1 in forming NXF1/NXT heterodimers [7]. NXT2 has two alternative splicing variants of transcripts encoding p15-2a and p15-2b proteins that differ in the first exon [7]. To date, the function of NXT2 variants has not been elucidated.

We previously generated cloned zebrafish from long-term cultured fibroblast cells infected with a retrovirus expressing GFP reporter gene [21]. We bred the GFP transgene to homozygosity and found that one of the cloned fish lines bore a recessive embryonic lethal mutation, suggesting that viral insertion is the cause of the cardiac defect. More interestingly, the mutant embryos have reduced circulation, cardiac edema and aberrant heart valve formation after three days of development. Sectioning of homozygous mutant embryos revealed that the ventricular wall is thinner and the space between the myocardium and endocardium is enlarged. Molecular analysis of these mutants revealed that *NXT2 *was disrupted by the viral insertion. Whole-mount RNA *in situ *hybridization detected *NXT2 *expression in the heart as well as other tissues. RT-PCR analysis suggested that *NXT2 *has multiple alternative mRNA splicing forms and intriguingly, one of them is significantly reduced in mutant embryos. Morpholino knockdown of this specific splicing form reproduced the mutant phenotypes. Taken together, our data demonstrate that NXT2 has a critical role in the morphogenetic integrity of the embryonic heart in zebrafish, and provide the first functional study of NXT2 in a vertebrate animal species.

## Results

### Identification and characterization of a cloned zebrafish exhibiting heart defects

**Figure 1:**
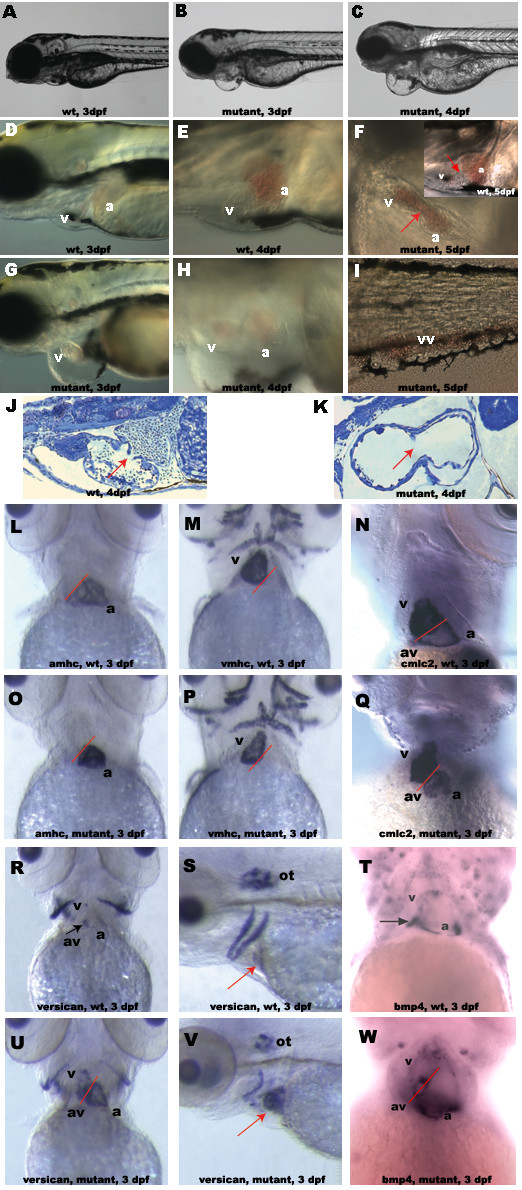
Characterization of NXT2 mutant phenotypes. At 3 dpf, NXT2 mutants show pericardial edema (compare B and G to wild-type embryos A and D). By 4 dpf, both atrium and ventricle of NXT2 mutants show significant dilation (compare H to wild-type embryo E) with pericardial sac enlargement (C and H). At 5 dpf, NXT2 mutant heart appears to be lacking atrioventricular boundary (F, arrow) and shows blood accumulation in both cardiac chambers in enlarged heart sac (F) and /or peripheral circulation (I, tail vessel) whereas the wild type heart with a normal atrioventricular boundary (arrow) resides in a narrowed cardiac sac (inset in F). Sections of wild-type (J) and NXT2 mutant (K) hearts at 4 dpf show that ventricular wall of NXT2 mutant embryos is thinner and a space separating myocardium and endocardium in atrium is visible. In addition, we often observed mutant embryos lacking valve structure (arrows). RNA whole mount *in situ *hybridization analyses of myocardial differentiation in wild-type (L, M, N, R, T) and NXT2 mutant (O, P, Q, U, W) at 3 dpf. All ventral view, anterior to the top except S and V, which are lateral view, anterior to the left. Solid red line marks the site of the boundary between the ventricle (v) and the atrium (a). NXT2 mutants show normal expression of cardiac chamber-specific markers *amhc *(L and O), *vmhc *(M and P), *cmlc2 *(N and Q). At 3 dpf, *versican *(R and U) and *bmp4 *(T and W) are restricted to the atrioventricular boundary in wild-type embryos (black arrows), but, are diffusely expressed in both cardiac chambers in NXT2 mutants. *Versican *expression pattern in otoliths is not changed in mutant embryos (S and V, red arrows: heart). vv: veinous vessels; a: atrium; v: ventricle; av: atrioventricular boundary; ot: otoliths. wt: wild type; mutant: *zNXT2 *homozygous mutant embryos; dpf: days post fertilization.

Cloned zebrafish were obtained by nuclear transfer [21] from long-term cultured cells that were infected with retrovirus [22] containing a GFP reporter gene driven by the *Xenopus ef1 alpha *promoter. It has been shown that retroviral integration can cause mutations in zebrafish [23]. We therefore bred all of the cloned fish lines to homozygous for GFP to screen for abnormality. One of these lines exhibited specific heart defects. The mutant phenotype co-segregated with proviral GFP expression (n > 2000), suggesting that the heart defects were caused by disruption of a functional gene by retroviral insertion. The mutant embryos were first distinguishable from their wild-type siblings by slightly dilated heart, slow heart rate, circulation reduction and mild pericardial edema after 3 days of development (Fig 1A, B, D and 1G). At this stage, cardiac function assays showed that the atrium and ventricle both contracted rhythmically in a normal atrio-venticular sequence. But, the heartbeat rates of mutants (134.3 ± 11.1 per minute, n = 72) were statistically significantly (P < 0.01) slower than that of their wild-type siblings (154.7 ± 8.7 per minute, n = 42) and the significant difference remained at 4 days post fertilization (dpf) (mutant 134 ± 12.8 per minute, n = 37 *vs *wild type 169 ± 4.7 per minute, n = 23; P < 0.01). Furthermore, while the wild-type embryos (Fig 1E) develop a narrow heart with forward blood flow through the embryo, both cardiac chambers are dilated in the enlarged pericardial sac (Fig 1C and 1H) and blood cells regurgitated between ventricle and atrium, resulting in either pooling the blood cells in two heart chambers or peripheral veins of mutant embryos at 4–5 dpf (Fig 1F, H and 1I). By 5 dpf, the mutant hearts are stretched to a long tubular structure (Fig 1F). Pericardial edema became prominent, circulation was completely terminated and severe edema appeared in the whole abdominal cavity, leading to necrosis in fin, muscle, skin and other tissues (data not shown).

The reduced circulation and regurgitation defects may be the results of abnormalities in cardiac contractibility, differentiation of cardiomyocytes or the formation of cardiac valve. To further investigate these possibilities, we analyzed histological sections of wild type and mutant embryos. Normally, by 2 dpf, the ventricular myocardial cells thicken and become the primary pumping force of the heart. We found that in 4 dpf mutant embryos, the ventricular wall remains thin and the space between myocardium and endocardium was diminished in the ventricle but enlarged in the atrium compared to that of wild type embryos (Fig 1J and 1K). In addition, we frequently observed mutant samples lacking cardiac valve structure. In 5 dpf mutant embryos, the ventricular wall remained thinner and cardiac chambers were severely dilated (data not shown). These data suggested that myocardial structures were disturbed in mutant embryos during early development.

Various heart chamber-specific gene expressions demarcate vertebrate heart compartments and play an important role in regulating critical steps of chamber formation. To understand whether the identified mutation affects formation of specific chambers and morphogenetic movements of dynamic cardiomyocycte, expression of chamber-specific genes including *Vmhc, Amhc *and *Cmlc2 *was examined. As shown in Fig 1L–Q, no difference was detected in expressions of *Amhc *(L and O), *Vmhc *(M and P) and *Cmlc2 *(N and Q) between wild-type (L, M and N) and mutant (O, P and Q) embryos at 3 dpf (same results were obtained at 4 and 5 dpf, data not shown), suggesting that the mutated gene is not required for the initial chamber-specific differentiation of cardiomyocytes, in which *Amhc, Cmlc2 *and *Vmhc *play important roles [24-27].

Epithelial-to-mesenchymal transition (EMT) plays a critical role in atrio-ventricular valve formation. In the zebrafish, this process begins at 33 hpf, when the myocardial cells at the atrio-ventricular boundary induce the adjacent endocardial cells to undergo EMT[28,29]. *Bmp4 *and *versican (cspg2) *are genes involved in cardiac valve formation. Coincidently, their cardiac expressions become restricted to the myocardial cells at the valve forming region at the same time as the formation of the prevalvular cushions, suggesting that they are thereby markers for the differentiation of the myocardial cells in the valve forming region and misexpression of these genes may indicate defects in valve formation[27,28]. As shown in Figure 1 (R-W), expression of *versican *and *bmp4 *in mutant embryos (Fig 1U, 1V, and 1W) was broadly up-regulated and expanded throughout the two heart chambers at 3 dpf at which stage their expression should be restricted to the atrioventricular boundary as in wild-type embryos (Fig 1R, S and 1T). Meanwhile, no difference of *versican *expression (Fig 1S and 1V) was detected in the otoliths of developing ears in wild-type and mutant embryos [30,31], suggesting that the abnormal *versican *expression pattern is due to heart defects observed in these mutant embryos other than general developmental delay.

### NXT2 is responsible for heart defects

**Figure 2:**
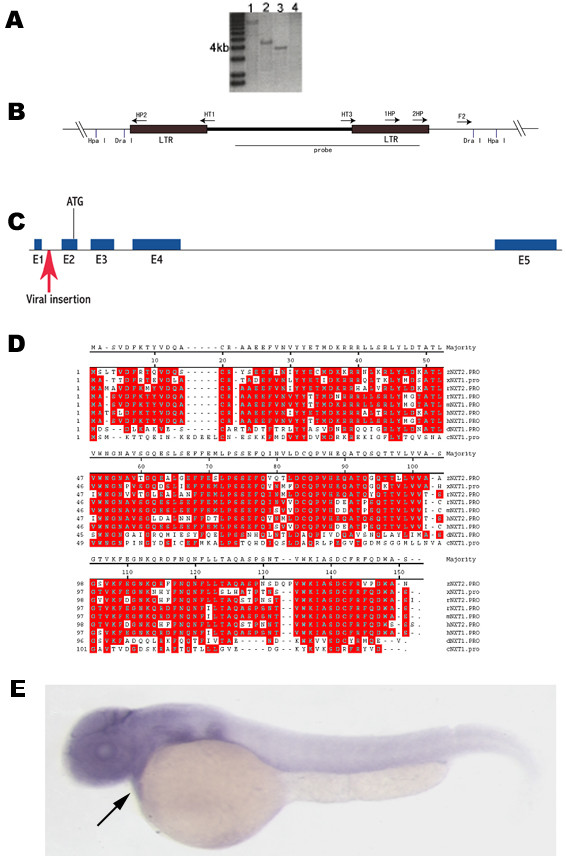
Identification of *NXT2 *as a candidate gene responsible for the heart defect. Southern blot analysis shows that a 4.3 kb (digested by Hpa I; A, lane 2) and a 3.8 kb (digested by Dra I; A, lane 3) are detectable using a specific viral DNA probe (B). Lanes 1 and 4 (A) are positive and negative control, respectively. DNA fragments corresponding to 3.8 kb / Dra I and 4.3 kb / Hpa I restriction were gel-purified and used for inverse PCR to isolate junction genomic sequences with primers described in (B) and Methods. This resulted in identification of NXT2 gene, which consists of five exons (C) and encodes 143 putative amino acids (D). The provirus is inserted within the first intron (red arrow) upstream of the putative initiation codon ATG in exon 2 (C). Alignments of protein sequences from various organisms revealed that zebrafish NXT2 shares 73.9%, 72.5%, 71.6%, 70.2%, 70.9%, 68.8%, 43.6% and 34.6% amino acid homology with that of rat NXT2, human NXT2, human NXT1, rat NXT1, mouse NXT1, *Xenopus *NXT1, *Drosophila *NXT1 and *C. elegans *NXT1, respectively (D). Identical residues are shown in red boxes. Zebrafish *NXT2 *is ubiquitously expressed at 2 dpf as shown by RNA whole mount *in situ *hybridization (E, arrow points to heart). E: lateral view, anterior to the left. zNXT2: zebrafish NXT2; hNXT1: human NXT1; hNXT2: human NXT2; mNXT1: mouse NXT1; rNXT1: rat NXT1; rNXT2: rat NXT2; cNXT1: *C. elegans *NXT1; dNXT1: *Drosophila *NXT1 and xNXT1: *Xenopus *NXT1.

Of the 2000 analyzed mutated embryos, the heart defect always co-segregate with the proviral GFP expression, suggesting that the viral insertion causes the observed heart phenotypes. By analyzing the genomic DNA by Southern blot, we found that there is a single viral insertion in this fish line (Fig 2A and 2B). A 1071 bp genomic sequence flanking the viral insertion was isolated by inverse PCR and used to search EST databases, leading to the identification of a candidate gene encoding a 143 amino acid protein that shares high homology with that of NXT1 and/or NXT2 from human, mouse, rat, *Drosophila *and *C. elegans*. By comparing genomic structures of *NXT1 *and *NXT2 *genes from these species, we noticed that *NXT1 *was encoded by a single exon whereas *NXT2 *gene contains multiple exons, which is similar to our candidate gene (Fig 2C). Based on similarities in protein sequences (Fig 2D) and genomic organizations, we concluded that our candidate gene represents a homolog of *NXT2 *rather than *NXT1*and named it *zNXT2*.

To determine the expression pattern of *zNXT2*, we carried out RNA whole mount *in situ *hybridization with its sense and antisense RNA probes. It was found that *zNXT2 *was ubiquitously expressed during early embryogenesis but clearly detectable in heart at 2 dpf (Fig 2E).

### zNXT2a is the alternatively spliced transcript required for heart function

**Figure 3:**
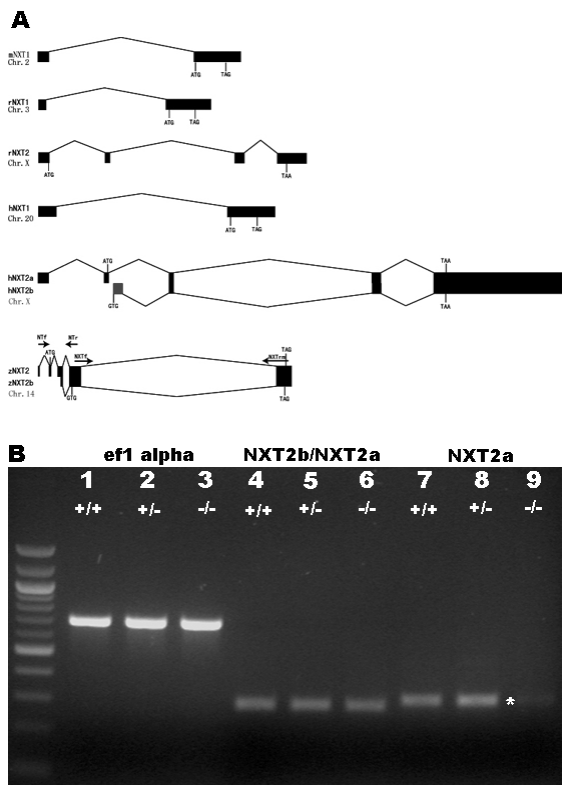
Alternative splice variants of the zebrafish *NXT2*. **A: **Solid black boxes represent exons, lines represent introns. Initiation codons and stop codons are indicated in capitalized letters. Solid blue box represents an exon of human NXT2 splice variant NXT2b, with GTG as initiation codon. The zebrafish NXT2 splice variant NXT2b is indicated below zebrafish NXT2 with GTG as initial codon. Primers NTf/NTr and NXTf/NXTrm (horizontal arrows) were used for PCR amplification to identify splicing variants of zNXT2. chr.: chromosome or linkage group. **B: **RNA expression profile of zebrafish *NXT2 *splice variants by RT-PCR. Equal amounts (2 μg) of total RNA, isolated from wild-type embryos (lanes 1, 4, 7), NXT2 mutant embryos (lanes 3, 6, 9) and their heterozygous siblings (lanes 2, 5, 8), were used for RT-PCR analyses. Lanes 1–3 refer to PCR products of endogenous eflα gene as positive control, lanes 4–6 represent products of primers NXTf/NXTrm, and lanes 7–9 indicate PCR products of primers NTf/NTr. RNA transcription level of *zNXT2*, represented by NTf/NTr in lane 9 (indicated by an asterisk), is remarkably reduced in mutant embryos, compared with that of their heterozygous and wild-type sibling embryos in lanes 8 and 7. No significant difference is detected among different genotypic embryos by NXTf/NXTrm in lanes 4, 5 and 6. -/-: *zNXT2 *homozygous mutant; +/-: heterozygous sibling;+/+: wild type sibling; ef1 alpha, NXT2a and NXT2b: transcripts detected by appropriate primers.

Search for zNXT2 homologues revealed that one copy of *NXT *gene exists in *Drosophila, C. elegans *and mouse whereas two copies are present in rat (rNXT1 and rNXT2) and human (hNXT1 and hNXT2) genomes, respectively (Fig 3A). In humans, two alternative splicing variants of *NXT2 *(*NXT2a *and *NXT2b*) were identified in EST database, which were further confirmed by cloning and sequencing the corresponding cDNAs [7]. In order to determine if *zNXT2 *also has multiple alternative RNA transcripts, 5' RACE analysis was performed [32] and the PCR products were sequenced. From 19 sequences obtained, 6 represented the original *zNXT2 *encoding 143 amino acid protein as mentioned above (now named as *zNXT2a*), 2 lacked the third exon, and 11 did not have the first two exons and part of third exon. The last variant, now named *zNXT2b*, appeared to be transcribed from the third exon and started at the putative codon GTG (Fig 3A). The same observation has also been made in humans in that the second splicing variant *NXT2b *starts from the putative initiation codon GTG [7]. The putative zNXT2b protein contains most of the functional domains of zNXT2a, since they share the last two identical exons.

To determine which transcript is responsible for the mutant phenotype we compared *zNXT2a *and *zNXT2b *mRNA expression level in mutant and wild type embryos. Two sets of primers, NTf/NTr for the first and the fourth exon to detect *zNXT2a *and NXTf/NXTmr for the fourth and the fifth exon to detect both *zNXT2a *and *zNXT2b*, were designed for RT-PCR analysis (Fig 3). As shown in Fig 3B, *zNXT2a *expression level was dramatically reduced in homozygous mutant embryos (indicated by an asterisk) compared to their wild-type or heterozygous siblings whereas no difference of mRNA levels was observed when both *zNXT2a *and *zNXT2b *were detected using NXTf/NXTmr primers. Taken together, these data suggested that loss of *zNXT2a *transcripts might be responsible for this mutant phenotype.

### Blocking zNXT2a activity by morphlino phenocopies the mutant heart defects

**Figure 4:**
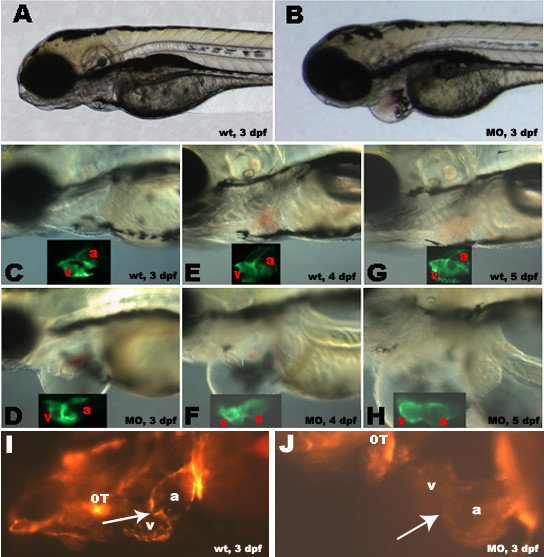
Morpholino antisense knockdown of *zNXT2 *in transgenic zebrafish embryos expressing cmlc2-GFP and flk-RFP. zNXT2 knockdown causes pericardial edema (compare A, C, E, G with B, D, F, H), abnormal relative positions of two chambers and chamber dilation (compare insets in C-H showing CMLC2 promoter driven GFP expression patterns in myocardium of atriums and ventricles). Observation of endothelial cells at the atrioventricular boundary in living *flk-1*- RFP transgenic embryos allowing visualization of cardiac valve formation (I and J). Clustering of endothelial cells was clearly visible in the cardiac valve region in wild-type embryos (I, arrow) whereas injection of *zNXT2 *morpholino caused a failure of clustering of endocardial cells at the atrioventricular boundary (J). All embryos are lateral view, anterior to the left. a: atrium; v: ventricle; OT: outflow tract; A, C, E, G: uninjected wild-type embryos; B, D, F, H: morpholino-injected embryos. wt: wild type; dpf: days post fertilization; MO: zNXT2 morpholino.

Morpholino technology is a powerful tool for blocking translation of specific mRNA transcripts and has been extensively used in zebrafish [33-35]. To further verify that zNXT2a was responsible for the observed heart defects, an antisense morpholino oligo specific to this transcript was injected in wild type embryos at one to four-cell stage. Analysis of more than 500 embryos from multiple injected clutches showed that approximately 80% of injected embryos developed the same heart defects as that in the virus-induced mutant embryos (Fig 4A–H), including pericardial edema, slightly dilated heart, slow heart rate and reduced circulation. Mock-injections have no effect on heart development in control embryos (data not shown).

The ability to use morpholinos to reproduce *zNXT2a *mutant phenotypes allowed us to confirm cardiac defects observed in living mutant embryos (Fig 1) through using live transgenic zebrafish embryos expressing GFP in specific cell lineages. First, cardiac myosin light chain 2 (CMLC2) promoter driving GFP transgenic zebrafish [26] embryos were used to determine development of two heart chambers. As shown in Fig 4, heart chambers of morpholino injected embryos at 3 dpf exhibited pericardial edema and dilated heart compared with that of wild-type embryos (Fig 4A, B, C and 4D). In 4–5 dpf morpholino injected embryos, cardiac chambers became abnormally stretched and relative positions of two chambers were abnormal (Fig 4F and 4H) whereas wild-type embryos had heart chambers in a narrowed pericardial sac (Fig 4E and 4G). This finding is consistent with the previous results described based on viral insertional mutated embryos (Fig 1).

We further examined endocardial morphology at the atrioventricular valve region using receptor tyrosine kinase *flk-1 *promoter driving RFP transgenic zebrafish embryos. Previously, tie-2 GFP transgenic zebrafish has been used to address this issue. Similar to *tie-2*, *flk-1 *also plays a crucial and unique role in endothelial cell differentiation and vascular morphogenesis, including vessels and heart endocardium [36,37]. In uninjected wild-type embryos, endocardial cells cluster at the atrioventrivular boundary and form a ring shape structure at the valve forming region (Fig 4I). However, more than 70% of the *zNXT2a *morpholino injected *flk-1*-RFP transgenic embryos (n = 129) failed to form this cluster (Fig 4J), suggesting that *zNXT2 *is required for this early endocardial morphogenetic event. Together with RNA whole mount in situ data of *bmp4 *and *versican*, these results indicate that cardiomyocyte differentiation and thickening of the endocardium at the atrioventricular boundary in the mutant embryos were significantly impaired, resulting in abnormal valve formation.

## Discussion

### Ventricular myocardial thickening and atrioventricular valve are impaired by disruption of zNXT2a

In zebrafish, between 24 and 48 hpf, the linear heart tube gradually bends at the boundary of two chambers to create an S-shaped loop. Meanwhile, clustering of endocardial cells as a ring structure at the atrioventrocular boundary by 33–37 hpf initiates the first step of the formation of cardiac valve [27,28,31]. While the heart is pumping, differentiation and morphogenesis continue, which includes remodeling of the cardiac valve to completion, finally building on a linear heart tube to produce an adult heart.

Sectioning of *zNXT2a *homozygous mutant embryos revealed that the ventricular wall is thinner as early as 3 dpf (data not shown, but see Fig 1 for 4 dpf data). Decreased cell proliferation and/or increased apoptosis may cause such a defect. Since the thinner myocardial wall appears very early during development we prefer a model that the under-developed ventricular wall is due to a deficiency in myocardial cell proliferation. Alternatively, this could result from abnormally tight intercalation of ventricular cells with the same numbers of cells in the mutant embryos as previously described [38]. In regard to the atrioventricular valve defect, it has been reported that endothelial shear stress (blood flow) plays a critical role in endocardial cushion by using special surgical experiments in zebrafish embryos [39]. In contrast, other researchers [40] argued that reduction in myocardial function is primarily responsible for the defect in atrioventricular cushion development, which was observed in *sih *(no early heartbeat) and *cfk *(cardiac dilation and no early blood flow) mutant embryos. In *zNXT2 *mutant embryos, the earliest observable defects are reduced heart rate, dilated cardiac chambers and a thin ventricular myocardial wall. Heart morphology and chamber specification ascertained by expression of *Amhc, Vmhc *and *Cmlc2 *are all normal at this stage in mutant embryos (Fig 1 and 4, data not shown by 2 dpf). Later on from 3–4 dpf, the atrioventricular valve was aberrant and the relative position of atrium and ventricle was perturbed, as revealed by sectioning analysis, expression of *versican *and *bmp4 *(Fig 1) and morpholino knockdown in CMLC2-GFP and flk-GFP transgenic embryos (Fig 4), respectively. Additionally, injection of a higher dose (approximately 10 ng per embryo) of *zNXT2a *morpholino into embryos causes defective endocardial cushion formation as early as 2 dpf, as illustrated by a failure of endocardial clustering at atrioventricular boundary (data not shown). This indicates that valve malformation resulted by interruption of *zNXT2a *might be either a primary defect or a defect due to disturbed function of chambers at later stage. In addition, our data implies that the mutant cardiac valve malformation is either an independent event or caused by lacking of myocardial cell proliferation or differentiation. The later situation will be consistent with the observation by Bartman et al (2004). Nonetheless, our findings establish that zNXT2 has a critical role in driving cardiac valve formation to final completion.

### zNXT2a is the mRNA transcript variant responsible for heart defects

As in humans, *zNXT2 *has multiple alternative splicing mRNA transcripts with two distinct start sites (ATG and GTG) (Fig 3A). It is possible that different *zNXT2 *splicing variants are differentially expressed in different tissues. Moreover, *zNXT2b *starting from GTG may represent a major transcript because of its higher copy numbers in the amplified cDNA pools (11 out of 19 sequenced samples) whereas *zNXT2a *starting from ATG might be enriched in heart. The biological significance of such a variation in *NXT2 *transcription has not been addressed. Our studies demonstrated that the alternatively spliced transcripts might have specific functions in different tissues during embryonic development and organogenesis. Specifically, *zNXT2a *appears to be required for heart development in zebrafish. Viral insertion in the intron immediately upstream of the exon containing ATG caused specific knockdown of *zNXT2a *transcription in mutant embryos whereas other transcripts such as *zNXT2b *appear normal. Injection of a morpholino against *zNXT2a *can phenocopy heart defects, confirming that zNXT2a protein is the main factor required for zebrafish heart development. Furthermore, when the morpholino dose was increased, more injected embryos developed pericardial edema one day earlier than the insertional mutants of *zNXT2 *(data not shown). This suggests that our *zNXT2a *insertional mutant may be a hypomorphic allele due to insertion of the viral vector into a non-coding region of the locus. With morpholino data it is clear that *zNXT2a *is directly implicated in heart development that requires its sufficient expression activity.

### Nuclear transport factors are implicated in tissue-specific biological functions

Nuclear protein or RNA transporting has previously been regarded as a general house keeping function for all biological systems. However, increasing evidence suggest that this can be a highly regulated process for specific biological functions with specific RNA targets. In *Drosophila*, studies demonstrated that homolog of NTF2 (DNTF2) is required for nuclear translocation of the Rel proteins Dorsal, Dif and Relish into the nucleus. These three Rel proteins are activated in response to immune challenges and imported into the nucleus upon microbial challenges, which further induce and regulate transcriptions of anti-microbial peptides attacin, cecropin, defensin, metchnikowin, diptericin and drosomycin. Disruption of DNTF2 leads to impairment of nuclear import of the Rel proteins and thus immune response is impaired in NTF mutants compared with wild type [41,42]. NXF5, a member of the nuclear export factor NXF family, binds to RNAs as well as to NXT members for RNA nuclear export. In a male patient with a syndromic form of mental retardation, a nonfunctional NXF5 was identified, which is split by the breakpoint situated in the 5' UTR between exons 1 and 2 of *NXF5*. This suggests that interruption of the gene NXF5 might result in the disease phenotypes associated with syndromic mental retardation including short stature, general muscle wasting, and facial dysmorphism by deranging the export or transport of specific mRNAs [43,44].

Formation of an NXT1 (or NXT2) and TAP (NXF1) heterodimer is critically required for mRNA export from the nucleus via TAP/NXT1 pathway [9,10,13]. Earlier experiments demonstrated that NXT1 is essential in Crm1-mediated nuclear export of proteins, in which NXT1 directly binds to export receptor Crm1 and RanGTP, and improves the release of cargos into the cytoplasm after translocation through the NPC [15]. Very recently, it has been reported that CRM1 is involved in export of specific subsets of cellular RNAs, which include *tra-2 *mRNA participating in sex cell fate determination in *C. elegans *and *IFN-a1 *mRNA generated upon viral infection to human cells [45,46]. However, little is known about how their cofactor NXT1 (or NXT2) plays a role in a biological pathway. Our finding provides the first evidence that an alternative splicing form of nuclear transport factor NXT2 is specifically implicated in heart development of zebrafish. For future studies, identification of specific RNA targets and elucidation of the molecular mechanism underlying NXT2-mediated exporting of these targets, either mRNA or micro RNA, in heart should be the focus. In addition, this study might provide the basis for some inheritable cardiac valve diseases in human. It should be a reasonable step to determine genetic linkage relationship between a NXT2 locus and defined cardiac diseases.

## Conclusion

We demonstrated that disruption of *NXT2 *gene, a member of NXT (nuclear export factor) family, is responsible for heart-specific defects in a mutant zebrafish generated by a retroviral insertion introduced into the genome by animal cloning technology. Our study suggests that NXT2 has critical roles required for myocardial cell differentiation and formation of cardiac valve at the atrioventricular boundary. Since NXT2 is highly conserved between fish and man it is conceivable that it may also play a role in mammalian heart development and might be involved in cardiac diseases.

## Methods

### Mutant identification and characterization

We initially obtained 14 cloned zebrafish lines by nuclear transfer [21]. After breeding each line to homozygosity for the retroviral insertion that was introduced into the zebrafish donor nuclei as a traceable GFP marker, we identified one line with embryonic heart defects that co-segregate with the GFP expression of the viral construct.

Adult zebrafish and embryos were maintained and staged as previously described [47]. Homozygous mutant embryos were imaged using an Axiocam imaging system (Zeiss). For histological analysis, fixed embryos were dehydrated, embedded in plastic (JB-4, Polysciences Inc.), sectioned at 8 μm and stained with Toludine Blue.

### Identification of NXT2 as the candidate gene

Two rounds of inverse PCR were performed to isolate genomic DNA fragments immediately flanking the viral insertion based on the retroviral sequence [48]. For the first inverse PCR, genomic DNA from heterozygous mutant fish was restricted with Dra I. This generated an approximately 3.8 kb proviral-genome-containing genomic DNA fragment as revealed by Southern blot hybridization with a specific viral probe (Fig 2A and 2B). Using Geneclean III kit (Bio 101, Vista, California), a pool of genomic DNA containing this fragment was purified from the gel, then ligated into circular form and finally used as inverse PCR templates. Two sets of primers specific to viral -sequences were designed as follows:

HT1:5'-AATCCCGGACGAGCCCCCAAATGAAAGA-3' and HT3: 5'-ATAGAGTACGAGCCATAGTTAAAATAAA-3' (outer primers);

HP2: 5'-TTTCTTTGTTCCTGACCTTGATC-3' and 1 HP: 5'-AACCCCTCACTCGGCGCGCCAG-3' (inner primers). Templates were amplified for two rounds. The final PCR products were subcloned into the pCR2.1 vector (Invitrogen, Life Technologies) for sequencing. The obtained sequence was used for designing new primers for additional inverse PCR. Similarly, heterozygous genomic DNA was also digested with Hpa I, which generated a 4.3 kb fragment containing retroviral genome and a flanking sequence for PCR amplifications. Again, two rounds of amplification were carried out for the second inverse PCR, in which a set of primers (HP2: 5'-TTTCTTTGTTCCTGACCTTGATC-3'; 2 HP: 5'- TCGTGGTCTCGCTGTTCCTTGG-3') against the retroviral LTR region was used to enrich for DNA templates. Then the second round of PCR amplification was performed from the enriched templates with another set of primers HP2 and F2 (5'-GTGCATGGATAAGAAAAGACGGGTAACG-3', which is against the zebrafish genomic sequence flanking the proviral insertional locus) (Fig 2A and 2B). A total of 1071 bp genomic sequence was obtained and used to search for EST and genomic databases of zebrafish.

### Sequence analysis

To identify the mRNA coding sequence, the 1071 bp genomic flanking sequence was used to BLAST search against the zebrafish EST database. An EST (BI325953) was identified that shares identical sequence to the inverse PCR product and contains an open reading frame of 143 amino acids. Highly similar protein sequences from different species including human, mouse, rat, *Drosophila *and *C. elegans *were retrieved from cDNA databases by performing protein sequence similarity searches using the deduced zebrafish amino acid sequence. Multiple sequence alignments were constructed by using the Jotun Hein Algorithm Method (DNA Star Software). To predict the genomic structures of *NXT *homologues in various organisms, the BLASTN program for genomic sequences was employed based on cDNA and /or EST sequences.

### Morpholino knockdown

A morpholino was designed against ATG region of zebrafish *NXT2 *(5'-TCCACCGTTAAAGACATGACTGGTC-3', Gene Tools, LLC, Corrallis, OR). It was injected into one-cell to four-cell stage embryos at 3 μg/μl (2 nl per embryo) in 1 X Danieau solution as described by other researchers [35]. Different stages of injected embryos were imaged under a regular bright filter on a Zeiss microscope. Mock-injections using a morpholino targeting a pancreas-specific gene [49] or a morpholino with a random sequence at 4–6 ng per embryo was performed as controls.

### Identification of alternative splicing variants of zebrafish NXT2

To identify alternative splicing transcripts of NXT2, FirstChoice RLM-RACE Kit (Catalog #1700, Ambion) was employed to specifically amplify the 5' intact region of *zNXT2 *mRNA through its first strand cDNA using two pairs of 5' adaptor primers (outer primer: 5'-GCTGATGGCGATGAATGAACACTG-3'; inner primer: 5'-CGCGGATCCGAACACTGCGTTTGCTGGCTTTGATG-3') and 3' specific primers (NXTmr – outer primer: 5'- AAGCTCAGTTTGCCCAGTCT-3'; NTr – inner primer: 5'-TGGCAGTCAAGAGTTTGCAC-3') against the internal exons of the gene. RLM-RACE strategy is designed to amplify cDNA only from full-length, capped mRNA, usually producing a single band for a specific gene after PCR [32]. Total RNA isolated from 3-day wild-type embryos was used for 5' RACE experiments.

### Detection of mRNA transcripts by RT-PCR

To compare relative expression levels of *NXT2 *transcripts, total RNA samples were isolated from homozygous mutant embryos (-/-), their wild-type (+/+) and GFP positive normal (+/-) sibling embryos, respectively, which were sorted out based on GFP expression and heart phenotypes. For measuring mRNA levels, equal amounts of total RNA isolated from three types of embryos were subjected to first strand cDNA synthesis by reverse transcription under the same condition using M-MLV Reverse Transcriptase (Gibco, Invitrogen). And then, equal amounts of cDNA product were used for PCR amplification of targeted cDNA. Amplification of another cDNA, *ef1 alpha*, was used as a control. The following specific primers against *zNXT2 *coding sequences were designed across introns for this purpose: NTf, 5'-GACGGATGCTGGATTTGTTT-3'and NTr, 5'-TGGCAGTCAAGAGTTTGCAC-3'; NXTf, 5'-GAAACGCAGTCACTGGTCAA-3' and NXTrm, 5'-AAGCTCAGTTTGCCCAGTCT-3'. *ef1 alpha *primers were zEF1a-1: 5'-TCACCCTGGGAGTGAAACAGC-3' and zEF1a-2: 5'-ACTTGCAGGCGATGTGAGCAG-3'. Different cycle numbers (20, 25, and 29) of quantitative PCR amplification were employed to determine a threshold that produced detectable products on gel using the following program: 94°C, 3 minutes; 94°C 30 seconds, 60°C 40 seconds, 72°C 1.5 minutes; 72°C 10 minutes.

### Whole-mount RNA in situ hybridization

Sense and antisense digoxigenin-labeled RNA probes were generated from cDNA clones of the zebrafish *NXT2, amhc, vmhc, versican, cmlc2 *and *bmp4 *genes using a DIG/Genius 4 RNA Labeling kit (Boehringer Mannheim). Whole-mount RNA *in situ *hybridization conditions were as described by Jowett [50]. Images were obtained using an AxioCam digital camera (Zeiss) on Stemi SV11 Apo (Zeiss) dissection microscope.

## Authors' contributions

HH contributed to all aspects of the experimental data and drafting the manuscript. BZ performed some RNA in situ hybridization experiments. PAH carried out sectioning of mutants. JC provided discussions and edited the manuscript. SL designed the study, drafted and finalized the manuscript. All authors read and approved the final manuscript.
